# An integrated framework of personalized medicine: from individual genomes to participatory health care

**DOI:** 10.3325/cmj.2012.53.301

**Published:** 2012-08

**Authors:** Andrea W.M. Evers, Maroeska M. Rovers, Jan A.M. Kremer, Joris A. Veltman, Jack A. Schalken, Bas R. Bloem, Alain J. van Gool

**Affiliations:** 1 Department of Medical Psychology, Radboud University Nijmegen Medical Centre, Nijmegen, the Netherlands a.evers@mps.umcn.nl; 2 Department of Epidemiology, Biostatistics, and HTA, Radboud University Nijmegen Medical Centre, Nijmegen, the Netherlands; 3 Department of Gynecology, Radboud University Nijmegen Medical Centre, Nijmegen, the Netherlands; 4 Department of Human Genetics, Radboud University Nijmegen Medical Centre, Nijmegen, the Netherlands; 5 Department of Urology, Radboud University Nijmegen Medical Centre, Nijmegen, the Netherlands; 6 Department of Neurology, Radboud University Nijmegen Medical Centre, Nijmegen, the Netherlands; 7 Department of Laboratory Medicine, Radboud University Nijmegen Medical Centre, Nijmegen, the Netherlands; On behalf of the Task Force Personalized Medicine, Radboud University Nijmegen Medical Centre, Nijmegen, the Netherlands

**Abstract **Promising research developments in both basic and applied sciences, such as genomics and participatory health care approaches, have generated widespread interest in personalized medicine among almost all scientific areas and clinicians. The term personalized medicine is, however, frequently used without defining a clear theoretical and methodological background. In addition, to date most personalized medicine approaches still lack convincing empirical evidence regarding their contribution and advantages in comparison to traditional models. Here, we propose that personalized medicine can only fulfill the promise of optimizing our health care system by an interdisciplinary and translational view that extends beyond traditional diagnostic and classification systems.

Personalized medicine refers to the identification of risk factors and tailoring of management and treatment to the individual characteristics of each patient (1-3). This concept proposes to classify individuals into subpopulations that differ in disease etiology, development, and prognosis, susceptibility to a particular disease or their response to a specific treatment. Preventive or therapeutic interventions can subsequently be targeted to those who will benefit, to save costs for those who will not respond, and to decrease side effects for those who are likely to respond adversely. In addition, personalized medicine is also of utmost importance in other aspects of medicine, outside the scope of treatment. Based on genomic information, patients and families can for example obtain personalized information about the predicted disease development and optimize patient management. Also, they can learn about the risk of this disease occurring in further pregnancies and possibilities to prevent this by means of preimplantation genetic diagnosis. The term personalized medicine has become tremendously popular since US President Barack Obama, as a senator in 2006, introduced the Genomics and Personalized Medicine Act to facilitate the introduction of personalized medicine. Promising research developments in both basic and applied sciences, such as genomics (1-4) and participatory health care approaches (5-8) further boosted the common interest in personalized medicine among almost all scientific areas and clinicians.

However, the term personalized medicine is frequently used without defining a clear theoretical and methodological background. In addition, although conceptually attractive, to date most personalized medicine approaches still lack convincing empirical evidence regarding their contribution, and advantages in comparison to traditional models. Here, we propose that personalized medicine can only fulfill the promise of optimizing our health care system by an interdisciplinary, translational, and transdiagnostic view that extends beyond traditional diagnostic and classification systems. We anticipate the following challenges for these developments:

1. Since personalized medicine includes a broad interdisciplinary research area (including both basic and applied approaches), there is a need to develop a **translational research framework.** Such integrated interdisciplinary frameworks of personalized medicine are currently lacking, particularly in the more applied, clinical sciences, including concepts such as participatory health care, patient involvement, or patient-centeredness (1-3,5,6). Consequently, a coherent **framework for science, care, and management** has to be developed, in which tailored and individual based approaches are used beyond traditional diagnostics and classification methods.

2. Studies on the comparative effectiveness of personalized medicine in comparison with more traditional models are still scarce in almost all disciplines (1-3,7,8). However, to become the promised translational and interdisciplinary revolution of our health care system, the additional contribution and advantages of personalized medicine approaches (eg, tailored vs non-tailored approaches) have to be **systematically investigated and proven**.

3. Tailored approaches based on multidisciplinary cumulative data (including genetic, biomedical, and behavioral measures) from **experimental and observational databases** – proposed as a prerequisite for personalized medicine – are insufficiently available (1-3,7,8). Only these databases provide the size and flexibility needed to explore and confirm some of the relevant risk factors and subgrouping variables, which can help to individualize treatment decisions. In addition, the methodology of identifying relevant factors within these databases needs to be developed further.

To integrate these various approaches and to develop a common strategy for personalized medicine, the Radboud University Nijmegen Medical Centre has founded a Task Force with a broad research and clinical scope (including a range of methodological approaches, such as genomics, molecular biology, proteomics, epidemiology, and medical internet technology, as well as a range of medical and social disciplines, including urology, gynecology, neurology, and medical psychology). This interdisciplinary Task Force has the potential to develop an integrative framework of personalized medicine, by focusing on the following concrete goals:

1. Developing interdisciplinary and translational research networks for researchers, to develop **innovative strategies and health care approaches** in the area of personalized medicine;

2. Developing **risk-factor based models** for personalized medicine approaches that are no longer defined within existing classification and diagnostic systems, but are tailored to individualized risk factors of patients;

3. Assembling **databases of genetic, biomedical, and behavioral data** in cohort studies for testing generic and disease specific personalized medicine models;

4. Developing innovative methodological approaches of **testing specific personalized medicine models** from a genomic, social, epidemiological, and/or statistical view;

5. Conducting **interdisciplinary and translational research** into personalized medicine with a broad range of biomedical, chemical, technological, and social sciences;

6. Evaluating the additional **contribution and cost-effectiveness** of personalized medicine approaches (eg, tailored vs non-tailored care, patient involvement);

7. Implementing broad strategies of personalized medicine and participatory health care into **regular clinical care** (eg, personalized risk-factor based approaches of tailored care for various conditions).

Only when we are able to develop this integrated personalized medicine framework, personalized medicine can fulfill its promise of increased effectiveness, broad availability of personalized care approaches, and a stronger focus on participatory health care. The final goal is to develop a well-defined translational research and health care strategy that has benefits for each individual patient.
